# Interphase Engineering of Epoxy/Wool Biocomposites via Lignin Coating: Effects on Mechanical and Hygrothermal Performance

**DOI:** 10.3390/polym18212756

**Published:** 2026-11-05

**Authors:** Alessia Pantaleoni, Baljinder K. Kandola, A. Richard Horrocks, Irene Bavasso, Maria Paola Bracciale, Erika Palazzi, Carlos A. Fuentes, Khouloud Tilouche-Guerdelli, Jacopo Tirillò, Assunta Marrocchi, Fabrizio Sarasini

**Affiliations:** 1 Department of Chemical Engineering Materials Environment, Sapienza University of Rome, 00184 Rome, Italy; 2 Institute for Materials Research and Innovation, University of Greater Manchester, Bolton BL3 5AB, UK; 3 Luxembourg Institute of Science and Technology (LIST), L-4362 Esch-sur-Alzette, Luxembourg; 4 Department of Materials Engineering, University of Leuven (KUL), 3001 Leuven, Belgium; 5 Department of Chemistry, Biology and Biotechnology, University of Perugia, 06123 Perugia, Italy

**Keywords:** waste wool valorization, bio-based surface treatment, fiber–matrix interphase, water absorption, epoxy laminates, surface-engineered fabrics

## Abstract

This work has investigated epoxy composites reinforced with wool fabrics modified by an organosolv lignin coating deposited via a sustainable protocol. The lignin layer was introduced to tailor the fiber/matrix interphase, aiming to improve adhesion and reduce water-related degradation associated with the intrinsic hygroscopicity of wool. Laminates were manufactured by vacuum-assisted resin transfer molding (VARTM) and systematically characterized. Thermogravimetric analysis demonstrated improved thermal stability and higher char yield in lignin-coated composites. Mechanical tests revealed enhanced flexural strength (+21%) and modulus (+31%), together with a modest increase in interlaminar shear strength. SEM analysis of the fracture surfaces provided additional evidence of changes in fiber–matrix interfacial interactions. Low-velocity impact and DMA results further supported the role of the coating in improving load transfer and viscoelastic response. Water absorption tests indicated reduced equilibrium uptake and slower diffusion kinetics, highlighting the hydrophobic barrier function of the lignin layer. Collectively, these results show that the lignin coating acts primarily as an interphase modifier and water-barrier layer, enabling low-value wool fabrics to be upgraded into functional reinforcements for more durable epoxy biocomposites.

## 1. Introduction

The potential for reducing the environmental footprint of materials across manufacturing and engineering disciplines is driving the search for bio-based and sustainable solutions throughout their product life cycles [1]. Natural fiber-reinforced composites (NFRCs) have emerged as an established solution by combining weight reduction, energy savings, and end-of-life advantages with competitive specific mechanical performance [2]. The applications of NFRCs currently span sectors such as transportation, lightweight building components, and sports equipment, provided that durability and reproducible mechanical performance are ensured [3].

Among thermosetting polymers, epoxy resins remain a preferred choice because their dense crosslinked networks provide high stiffness and strength, as well as thermal and chemical stability, while limiting viscoelastic creep [4]. In this context, several studies have explored different natural fiber/epoxy systems and processing routes [5] with plant-based fibers being predominant due to their accessibility, affordability, and established transformation chains [2]. However, the broader adoption of epoxy-based NFRCs is still limited by two key issues: firstly, the poor interfacial compatibility between polar natural fiber surfaces and the hydrophobic epoxy matrix, which impairs wettability and load transfer [6]; secondly, the inherent tendency of the fibers to absorb moisture, which leads to swelling and microcracking, resulting in a loss of mechanical properties and dimensional stability [7,8,9,10].

However, natural fibers of animal origin have historically been less common in composites, owing to more demanding preparation processes and a less established supply chain compared to plant-based reinforcements. Their use has therefore been largely confined to specialized applications such as biomedicine, filtration and insulation [11]. Recently, however, interest has grown through the valorization of by-products and waste streams [12] and animal fibers explored have included keratin fibers from poultry by-products (such as chicken feathers [13]) and from the beauty sector (human hair [14]), together with wool, often recovered from shearing or processing waste [15]. According to a recent market analysis, the wool sector was valued at USD 42.029 billion in 2025 and is projected to expand to USD 56.95 billion by 2031, corresponding to a compound annual growth rate (CAGR) of 5.2% [16]. Roughly, while 90% of global wool production is used in the textile sector [17], large volumes of coarse and semi-coarse wool, often from meat-oriented breeds, do not meet the specifications of the apparel sector due to their high fiber diameters. This weak demand generates surplus and disposal challenges, sometimes leading to environmentally harmful practices banned in the EU [18,19]. This underutilized, low-value fraction represents an abundant feedstock for alternative applications, and composite production offers a route aligned with recycling and circular-economy objectives.

Epoxy composites reinforced with wool are of growing interest for their strong thermo-acoustic insulation properties [20,21], where, for example, hot-pressed wool/epoxy laminates with reduced thermal conductivity (≈30% vs. neat epoxy) have been reported [22]. Using a different processing route, Sharma et al. fabricated waste sheep wool/epoxy composites by VARTM using woven, non-woven, felt, and compressed-felt wool architectures, reporting that these systems combined low thermal conductivity with an increased glass-transition temperature (T_g_), indicating improved thermo-mechanical stability during service [12]. In addition, because keratin is rich in nitrogen and sulfur, which confer a propensity to generate char residues, wool has a high resistance to ignition and combustion [23] exemplified by a limiting oxygen index (LOI) value above 25% [24]. Thermoanalytical evidence also has indicated delayed degradation and increased char residue in wool/epoxy systems [22].

A well-known limitation of wool-based composites, however, is their hygroscopic nature. Wool fibers can reabsorb up to one-third of their weight in moisture and, within the amorphous fraction, up to ~45% of the dry mass (at ~25% crystallinity), which exceeds that of many plant fibers [25,26]. Such absorption mainly induces radial swelling and the plasticizing action of water weakens intramolecular bonds and reduces their mechanical properties [27]. Also during processing, fiber swelling and shrinkage reduce wettability and cause voids or debonding, while in service, interfacial water plasticizes the matrix and weakens load transfer as well as forming microcracks which encourage capillary absorption [28]. Consequently, reductions in modulus and strength, particularly in tension and flexure, along with losses in dimensional stability have been commonly observed [29].

As a consequence, some studies have investigated ways to limit moisture uptake by improving the wool–matrix interface. For example, José et al. reinforced wool/epoxy laminates with hydrophobic nanosilica and achieved mechanical gains and up to ~30% reduction in moisture content at 5.0 wt.% loadings, along with increased contact angle and slower diffusion [30]. Further work by Tsukada et al. [31], who grafted the hydrophobic monomer benzyl methacrylate (BzMA) on to wool fibers using ammonium persulfate, reported a markedly reduced equilibrium moisture regain, as the grafted polymer masked hydrophilic sites or acted as a barrier to diffusion, thus highlighting the potential of hydrophobic surface layers to mitigate swelling and diffusion-driven degradation in wool/epoxy systems.

These studies, although promising, remain limited to a few isolated examples and do not yet provide a systematic framework comparable to that available for lignocellulosic composites especially one that simultaneously reduces water uptake and enhances mechanical performance. In this context, the present work proposes the development of epoxy matrix composites reinforced with wool coated by a lignin-based polymeric layer.

Lignin is a renewable, highly branched aromatic macromolecule, available as a by-product of cellulose processing [32]. Its relative hydrophobicity, combined with the presence of phenolic and aliphatic hydroxyl groups, makes it suitable both as a physical barrier to water which is also reactive with the epoxy network [33]. For example, in hemp/epoxy laminates lignin has been used as a compatibilizer at low additions to improve stiffness/strength and impact performance [34]. Furthermore, Sun et al. showed that partially depolymerized and epoxidized lignin can be effectively incorporated into amine-cured epoxy networks to yield gains in modulus and fracture toughness [35]. Similarly, Feng et al. reported that waterborne epoxy coatings functionalized with quaternized lignin have displayed excellent dispersion, strong interfacial compatibility, and high resistance to corrosion, UV, and thermo-oxidative aging. The UV protection was ascribed to lignin’s conjugated π system (phenolics, ketones, aromatic rings), while radical scavenging contributed to aging stability [36].

While lignin has been extensively investigated as a modifier for epoxy matrices, its use as a surface-engineering agent for natural fiber reinforcements has remained largely unexplored. This study investigates the direct deposition of a lignin-based coating onto wool fabrics under mild and environmentally compatible conditions (i.e., in an aqueous medium, at low temperature, and using enzymatic catalysis), with the aim of modifying the fiber–matrix interphase in wool/epoxy composites.

The novelty of this study lies in translating this lignin-based surface treatment to epoxy/wool laminates as an interphase-engineering strategy, rather than as a bulk matrix modification. The aim is not to position low-value wool solely as a conventional load-bearing reinforcement, but to exploit the coated fabric as a waste-derived textile platform capable of improving water resistance and laminate-scale load transfer. This interphase-centered strategy represents a distinct alternative to conventional matrix-focused modifications.

In addition to performance gains, the approach supports circular resource utilization by valorizing two bio-based waste streams into functional composite materials, potentially lowering disposal costs of non-textile wool while also addressing one of the main durability limitations of wool/epoxy composites, namely water accessibility at the fiber–matrix interphase.

Rather than targeting high-performance primary structural composites, this approach is intended for semi-structural or non-primary structural applications where moderate mechanical requirements are combined with improved water resistance, damage tolerance, and the valorization of low-value bio-based feedstocks. Potential application areas include lightweight panels, interior components, protective casings, and insulating composite elements, where wool’s intrinsic thermo-acoustic and charring characteristics may provide additional functional value beyond conventional reinforcement.

## 2. Materials and Methods

### 2.1. Materials and Composite Manufacturing Process

#### 2.1.1. Materials

Sodium lignosulfonate (sodium salt, technical grade, average M_w_ ≈ 18,000 g/mol), laccase from *Aspergillus* sp. (Novozym^®^ 51003, liquid solution), acetate buffer components, hydrochloric acid (HCl), and Triton™ X-100 (octylphenoxypolyethoxyethanol, nonionic surfactant) were obtained from Merck KGaA (Darmstadt, Germany). Organosolv lignin (average M_w_ ≈ 3952 g/mol) was supplied by Tecnaro GmbH (Ilsfeld, Germany).

The wool fabric used in this study was supplied by Gledhill from Carr Reinforcements Ltd. (Burnley, UK), an independent weaver of high-performance fabrics. The fabric was a balanced 2/2 twill weave wool cloth (406 gsm, 730 mm width), with a density of 7.6 ends/cm and 7.3 picks/cm. Both warp and weft consisted of spun wool singles of 250 tex, resulting in comparable mechanical behavior in the two directions. The average fiber diameter, estimated from SEM micrographs of the untreated wool fabric, was 43.1 ± 11.8 μm (n = 25; range: 21.9–57.5 μm), indicating that the material can be classified as coarse wool.

#### 2.1.2. Wool Surface Modification

Wool fabrics were modified using a three-step protocol consisting of detergent scouring, treatment in a sodium lignosulfonate solution, and laccase-catalyzed oxidative polymerization of organosolv lignin on the fiber surface. Samples were first washed in a 20% (*w*/*v*) Triton^TM^ X-100 solution at 60 °C for 3.5 h, rinsed with deionized water, and dried overnight at 60 °C. The pre-cleaned material (46 g) was then treated in an aqueous sodium lignosulfonate solution (~9.2 g, ~0.51 mmol in 1.15 L; pH 3.0 adjusted with HCl) at 70 °C for 1.5 h, rinsed, and dried. This lignosulfonate pre-treatment was designed by adapting literature protocols on wool treatment with sulfonated lignin, in which acidic conditions were shown to favor lignin uptake/substantivity on wool fibers [37,38]. In the present work, this step was intended to introduce lignin-related phenolic moieties onto the wool fiber surfaces prior to the enzymatic treatment. After treatment, the fabrics were thoroughly rinsed to remove the loosely bound fraction.

The resulting lignosulfonate-treated fabrics were transferred to a 0.2 M acetate buffer solution (1.15 L, pH ~ 4.5) containing laccase (20 U mL^−1^; ~3.3 mL stock solution) and organosolv lignin (45.8 g, 11.6 mmol) and reacted at 60 °C for 2 h under continuous stirring to promote surface polymerization, obtaining organosolv lignin-treated fabrics (Wool_OL). This second step was selected on the basis of literature showing that laccase can operate under mild conditions and promote oxidation, coupling, grafting and/or polymerization of lignin-related phenolic systems [39,40]. These features make laccase a suitable tool for the sustainable surface modification of natural fibers and for improving interfacial interactions with polymer matrices [39,41]. Finally, samples were thoroughly rinsed with buffer and deionized water and dried at 60 °C.

The treatment conditions adopted in this study were selected following a preliminary optimization of the fiber functionalization process, in which different treatment parameters and technical lignins were investigated (not published). The selected treatment was chosen based on its overall performance, considering a combination of mechanical, moisture-resistance, UV-protection, antibacterial, and surface-morphology characteristics. The present study focuses on evaluating the suitability of the resulting modified fibers as reinforcement in epoxy-matrix composites, rather than on optimizing the functionalization process itself.

Fourier transform infrared spectroscopy (FTIR) spectra of untreated wool fabric (Wool_N) and organosolv lignin-treated wool fabric (Wool_OL) used for composite manufacturing are reported in the Supplementary Information as a qualitative verification of the surface treatment (Figure S1). Compared with Wool_N, Wool_OL showed spectral changes indicative of lignin deposition, including a downshift of the amide III band from approximately 1234 to 1221 cm^−1^, attributed to the overlap between the wool amide III vibration and lignin-related C–O/C–C stretching modes [42]. In addition, the treated fabric exhibited bands at approximately 1115 cm^−1^, characteristic of syringyl units in organosolv lignin [43], and at 1024 cm^−1^, associated with aromatic C–H deformation together with C–O/C–C stretching vibrations typical of lignin [44]. These spectral features provide qualitative evidence of the successful deposition of organosolv lignin onto the wool fabric surface. Scanning electron microscopy (SEM) micrographs of Wool_N and Wool_OL are also reported in the Supplementary Information (Figure S2). Compared with the untreated fiber surface, Wool_OL showed a rougher and less regular morphology, further supporting the presence of a lignin-based coating on the wool surface.

#### 2.1.3. Composite Manufacturing

The polymer-coated fabrics were employed as reinforcement for epoxy-based composites and prior to composite manufacture, coated fabrics were oven-dried at 60 °C for 24 h.

The epoxy resin (Bisphenol A EPILOK 60-822) and curing agent (amine-based CURAMINE 32-790 NT), both supplied by Bitrez Ltd. (Wigan, UK), were mixed in the ratio 100:38 by weight and degassed under vacuum. Three wool fabric plies, either untreated or lignin-treated, were infused via vacuum-assisted resin transfer molding (VARTM), cured for 24 h at ambient temperature, and post-cured for 8 h at 80 °C. The resulting wool/epoxy composites contained 28.4 ± 1.3 wt.% fabric reinforcement and had a thickness of 4.0 ± 0.1 mm. The experimental density of the composites was determined to assess potential differences between specimens reinforced with neat and lignin-coated fibers. The fiber volume fraction and void volume fraction were calculated based on the measured composite density and the relevant constituent properties [45]. For the composites reinforced with neat fibers, the experimental density was 1.195 ± 0.01 g/cm^3^, the fiber volume fraction was 25.90 ± 1.21%, and the void volume fraction was 2.79 ± 0.32%. For the composites reinforced with lignin-coated fibers, the corresponding values were 1.20 ± 0.01 g/cm^3^, 26.01 ± 1.19%, and 2.38 ± 0.72%, respectively. The measured densities and calculated fiber volume fractions are similar for the two composite systems, with only marginal differences between their mean values.

### 2.2. Composite Characterization

Fourier transform infrared spectroscopy (FTIR) analysis was performed on untreated wool fabric (Wool_N) and organosolv-lignin-treated wool fabric (Wool_OL) using a Bruker VERTEX 70 spectrometer equipped with a diamond ATR accessory (Ettlingen, Germany). Spectra were recorded between 400 and 4000 cm^−1^ at 4 cm^−1^ resolution, with 128 scans averaged for each spectrum.

Thermogravimetric analysis (TGA) was performed in accordance with ISO 11358 [46] using a TG 209 F1 Libra (NETZSCH-Gerätebau GmbH, Selb, Germany). Measurements were carried out under a nitrogen atmosphere from room temperature up to 800 °C at a heating rate of 10 °C·min^−1^. Each test was performed on approximately 20 mg of sample placed in alumina crucibles.

Dynamic mechanical analysis (DMA) was employed to evaluate the solid-state properties of the composites under dynamic loading conditions as a function of temperature. The storage modulus (E′), loss modulus (E″) and the damping factor tanδ (E″/E′) were determined. The experiments were conducted in a three-point bending configuration using a DMA 242 E Artemis (NETZSCH-Gerätebau GmbH, Selb, Germany), following ISO 6721 [47]. Specimens with dimensions of 60 × 10 × 4 mm were tested at a heating rate of 2 °C/min, in the temperature range from 35 °C to 150 °C, with a frequency of 1 Hz. The glass transition temperature (T_g_) was determined from the DMA measurements as the temperature corresponding to the maximum of the tanδ curve. The DMA specimens were cut from the composite plates with their longitudinal axis parallel to the warp direction of the reinforcement.

Flexural properties were assessed using a Zwick/Roell Z010 universal testing machine (ZwickRoell GmbH & Co. KG, Ulm, Germany). Three-point bending tests were performed according to ISO 178 [48] on specimens measuring 80 × 10 × 4 mm, with a crosshead speed of 5 mm/min and a span-to-thickness ratio of 16:1.

The interlaminar shear strength (ILSS) of the composites was evaluated following ASTM D2344 [49]. Short-beam three-point bending tests were performed on specimens with dimensions of 24 × 8 × 4 mm, using a span-to-thickness ratio of 4:1 as prescribed by the standard. The apparent ILSS was calculated from the maximum load at failure, normalized by the specimen width and thickness. This method provides an apparent laminate-scale assessment of the interlaminar shear behavior of fiber reinforced composites.

Charpy impact resistance was determined in edgewise mode following ISO 179–2 [50]. Unnotched specimens (80 × 10 × 4 mm, span length of 62 mm) were tested using a CEAST/Instron 9340 instrumented drop-weight tower (Instron, Pianezza, Turin, Italy) at an impact velocity of 2.90 m/s.

The surface morphology of untreated and organosolv-lignin-treated wool fibers, as well as the fracture surfaces of the composites, was examined by field emission scanning electron microscopy (FE-SEM, Mira3, Tescan Brno, s.r.o., Brno, Czech Republic). Prior to imaging, the samples were sputter-coated with a thin layer of gold.

Low-velocity impact behavior of the laminates was examined following the ISO 6603-2 [51] procedure. Tests were performed with a CEAST/Instron 9340 drop-weight tower fitted with a hemispherical striker (12.7 mm diameter). Impact energies of 2.5, 5.0, 7.5 and 10 J were obtained by adjusting the falling height of a 3.055 kg mass. Each specimen was secured between two rigid steel plates, leaving a circular unsupported region of 40 mm in diameter during the impact event. The tests were performed on square specimens measuring 6 × 6 cm^2^.

For each mechanical test, five specimens were tested, except for the low-velocity impact tests, for which three tests were performed at each impact energy.

Micro-computed tomography (µCT) scans were performed with an EasyTom system (RX Solutions SAS, Chavanod, France) operated at 70 kilovolts and 160 microamperes using a small focal spot generated by a lanthanum hexaboride (LaB_6_) filament. Radiographic projections were acquired at a frame rate of 0.75 frames per second, averaged over four frames, then reconstructed into isotropic voxels of 60.5 micrometers edge length. This voxel size defines the spatial resolution of the dataset; therefore, individual wool fibers (typically ~20–40 µm in diameter) and very fine microcracks are below the reliable resolvable limit. Consequently, the µCT analysis focused on mesoscopic damage features such as through-thickness cracks, localized crushing, and delamination rather than fiber-scale damage. The reconstructed µCT datasets were processed and visualized using Avizo software 3D (Thermo Scientific), version 2025.1.2.. For subsequent analysis, both radiographic Z-view projections (2D) and orthogonal tomographic slices, Z viewed along the X direction and Y viewed along the Z direction, were exported.

### 2.3. Water Absorption Testing and Diffusion Analysis of Composites

Water absorption behavior of the composites was assessed in accordance with ASTM D570 [52]. Prior to testing, specimens with dimensions of 10 × 10 × 4 mm were dried in an oven at 60 °C until a constant weight was reached. The conditioned samples were then immersed in distilled water maintained at 23 ± 2 °C. At predetermined intervals, the specimens were removed from the water, gently wiped with an absorbent cloth to eliminate surface moisture, and weighed with an analytical balance to the nearest 0.1 mg. Immediately after weighing, the specimens were returned to the water to continue immersion. The water uptake, expressed as the percentage increase in mass relative to the initial dry weight, was calculated using Equation (1):

(1) $$M(\%)=\frac{w_{t}-w_{0}}{w_{0}}\times 100$$

where w_t_ and w_0_ denote the specimen weight at time t and the initial conditioned weight, respectively. To evaluate the mechanism and extent of property degradation after water immersion, flexural tests were conducted on aged specimens that had been re-dried. The drying process was performed in an oven at 60 °C until constant weight was achieved.

The apparent diffusion coefficient of water in the composites was estimated according to Fick’s second law. Since the specimens were fully immersed with all edges exposed, the calculation was based on the surface-to-volume ratio (S/V) approach rather than on the simple slab geometry. In this method, the effective diffusion length *l_eff_* was expressed as *l_eff_* = 2/(S/V), where S and V denote the specimen surface area and volume, respectively. The diffusion coefficient D was then determined from the linear region of the sorption curve (M_t_ vs. √t), limited to values below 60% of the equilibrium uptake, using Equation (2) [53] and ASTM D5229 [54]:

(2) $$D=\frac{\pi }{16}\;\cdot {\left(\frac{l_{\mathrm{eff}}\cdot \;k}{\mathrm{M\infty}}\right)}^{2}$$

where M∞ is the equilibrium moisture content, and k is the slope of the initial portion of the absorption curve, determined according to Equation (3):

(3) $$k=\frac{M_{2}-\;M_{1}}{\sqrt{t_{2}}-\;\sqrt{t_{1}}\;}$$

Equation (2) is therefore the corrected diffusion coefficient for diffusion through the free edges according to the Shen and Springer correction factor [55,56,57].

Early stage sorption kinetics were quantified using the power-law model (Ritger–Peppas equation; Equation (4)):

(4) $$\frac{M_{t}}{M_{\infty }}=k\cdot t^{n}$$

where M_t_ is the moisture content at time (t), M_∞_ is the moisture content at saturation, and k and n are empirical constants. The parameter k is related to the sorption kinetics, whereas n characterizes the diffusion mechanism.

## 3. Results and Discussion

### 3.1. Water Absorption Behavior

**Figure 1:**
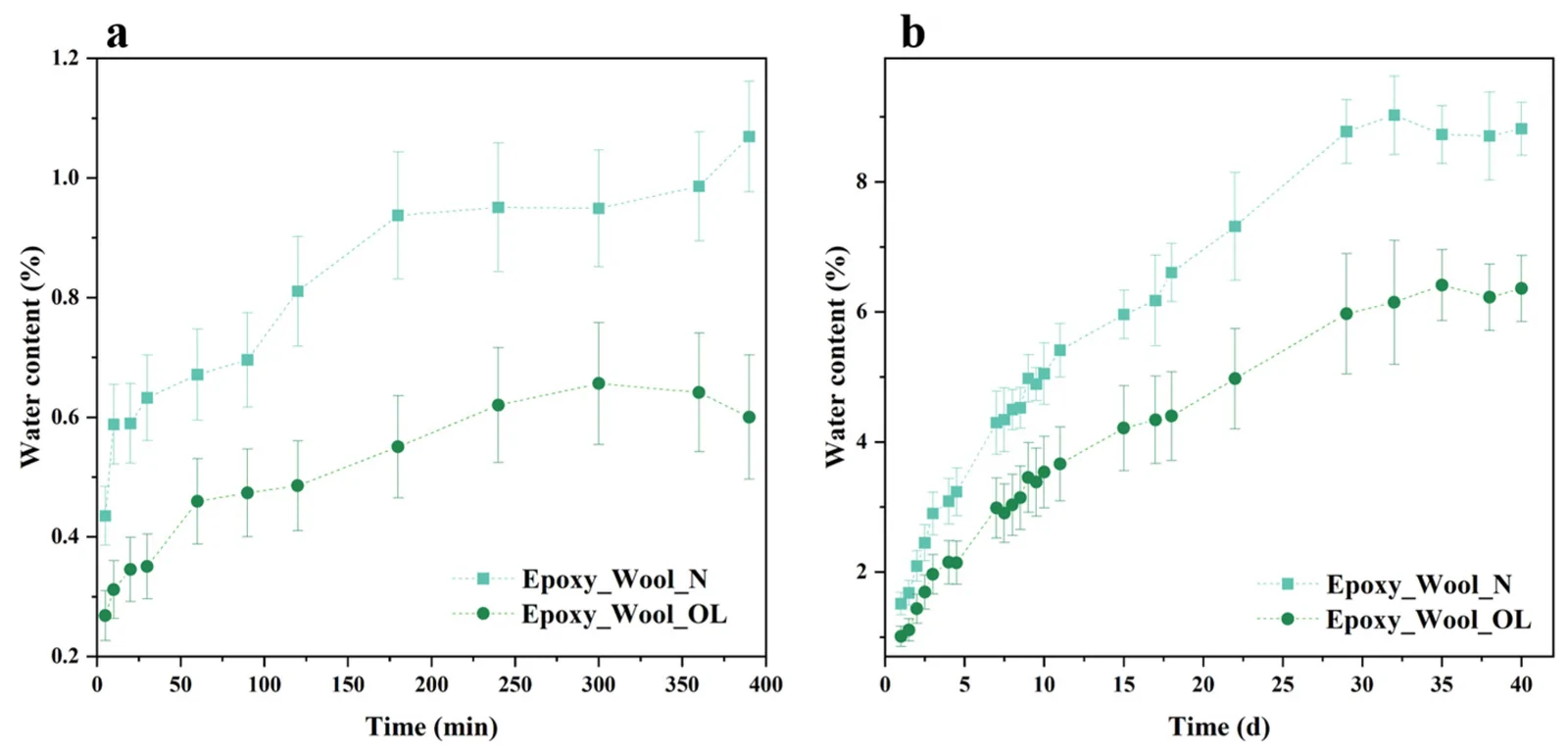
Water uptake kinetics of Epoxy_Wool_N and Epoxy_Wool_OL laminates, expressed as water content (%) versus immersion time after 390 min (**a**) and 40 days (**b**).

Since water absorption can significantly affect the mechanical properties and consequent durability of wool/epoxy laminates, the immersion behavior was examined. Figure 1 and Table 1 summarize the water uptake kinetics and the derived diffusion-related parameters for the control (untreated or neat wool) Epoxy_Wool_N and organosolv-coated wool Epoxy_Wool_OL composites.

The water absorption kinetics of wool-reinforced epoxy laminates highlight two distinct effects introduced by the organosolv lignin (OL) coating on the fiber surfaces within the wool fabric: (i) a reduction in the long-term water uptake and (ii) a slowdown of the effective transport during the early stages (Figure 1). The equilibrium uptake (M∞), reported in Table 1, decreases from 8.8 wt% for Epoxy_Wool_N to 6.4 wt% for Epoxy_Wool_OL, confirming the lower water accessibility of the coated system. The experimental data were fitted using the empirical power-law model in the initial-to-intermediate sorption region, corresponding to M_t_/M∞ < 0.6, and the corresponding fitting parameters and goodness-of-fit values are reported in Table 1. The fitting showed good agreement with the experimental data, with *R*^2^ values of 0.948 and 0.960 for Epoxy_Wool_N and Epoxy_Wool_OL, respectively. The model yields markedly sub-Fickian exponents, equal to 0.320 and 0.347, respectively, with a concomitant reduction in the rate constant k from 5.89 × 10^−3^ to 3.99 × 10^−3^ s^−n^. Values of n lower than 0.5 indicate that diffusion is not solely governed by the concentration gradient. This behavior can be attributed to the viscoelastic response of the epoxy network and fiber/matrix interphase, together with additional contributions from water-induced fiber swelling, the intrinsic hydrophilicity of wool, micro-voids or residual porosity, capillary transport, and interfacial water migration, which distort the ideal Fickian trend and result in a sub-Fickian regime, as widely reported for fiber/polymer laminates immersed in water [58]. Given the sub-Fickian behavior indicated by the fitted exponent n, the diffusion coefficients estimated using the conventional Fickian short-time approximation should be regarded as apparent diffusion coefficients. These values provide a comparative measure of the initial sorption kinetics but should not be interpreted as intrinsic Fickian diffusion coefficients.

Restricting the analysis to the initial stage, where M_t_/M∞ is proportional to √t, the Fickian fit provides a lower slope for the coated-fiber laminate, confirming that the apparent diffusion coefficient D is reduced in Epoxy_Wool_OL. Specifically, using the surface-to-volume ratio approach, the values are 5.38 × 10^−13^ m^2^/s for Epoxy_Wool_N and 3.74 × 10^−13^ m^2^/s for Epoxy_Wool_OL (Table 1). These values fall within the typical range reported for epoxy systems in contact with water, confirming the reliability of the data analysis and the physical consistency of the phenomenon [59]. In fiber reinforced laminates, water transport occurs primarily through diffusion in the polymer matrix, capillary flow through defects and microvoids, and transport along the fiber–matrix interphase. Interfacial debonding and moisture-induced relaxation may further contribute to water uptake. Improved interfacial adhesion and reduced connectivity of capillary pathways can therefore limit both the equilibrium moisture content and the effective diffusion coefficient derived from gravimetric measurements [60,61].

The organosolv lignin coating can achieve such an effect through several synergistic mechanisms. First, compared to the protein constituents of wool, mainly keratins rich in exposed polar groups (–COOH, –NH, –OH), lignin is relatively more hydrophobic and provides a coating with lower wettability and reduced vapor transmission. Similar barrier properties have been exploited in other contexts: for instance, Abbadessa et al. demonstrated that multilayer lignin coatings applied to packaging substrates significantly reduced water vapor and oxygen permeability [62], while lignin used as a coating on paperboard showed a marked decrease in water vapor transmission rate (WVTR) and an increased water contact angle, indicating an effective barrier function [63]. When applied as a sizing on fiber surfaces, lignin forms a continuous film with few highly polar groups exposed to water. Practically, the coating decreases the wettability of wool bundles, limits capillary imbibition within the fiber bundle, and reduces the number of active sites available at the epoxy interface. This translates into a lower accessible water content at equilibrium, resulting in a reduced M∞ value (Table 1), and a lower apparent diffusion coefficient.

Second, the reductions in M∞ and D may also be associated with changes at the fiber–matrix interphase. In laminates, capillary channels and connected porosity along the interface can provide preferential pathways for water transport; improving interfacial cohesion can therefore reduce both the uptake extent and the short-time transport rate [64]. This interpretation is supported by the mechanical results, dynamic mechanical analysis (DMA), and fracture-surface scanning electron microscopy (SEM) observations presented in the subsequent sections.

Overall, the organosolv lignin coating operates through a dual mechanism, namely it acts as a hydrophobic barrier that limits water accessibility and as an interfacial sizing that suppresses capillary transport along fiber–matrix pathways, thereby reducing both the rate and the extent of water uptake.

### 3.2. Flexural Behavior Before and After Water Immersion and Re-Drying

**Figure 2:**
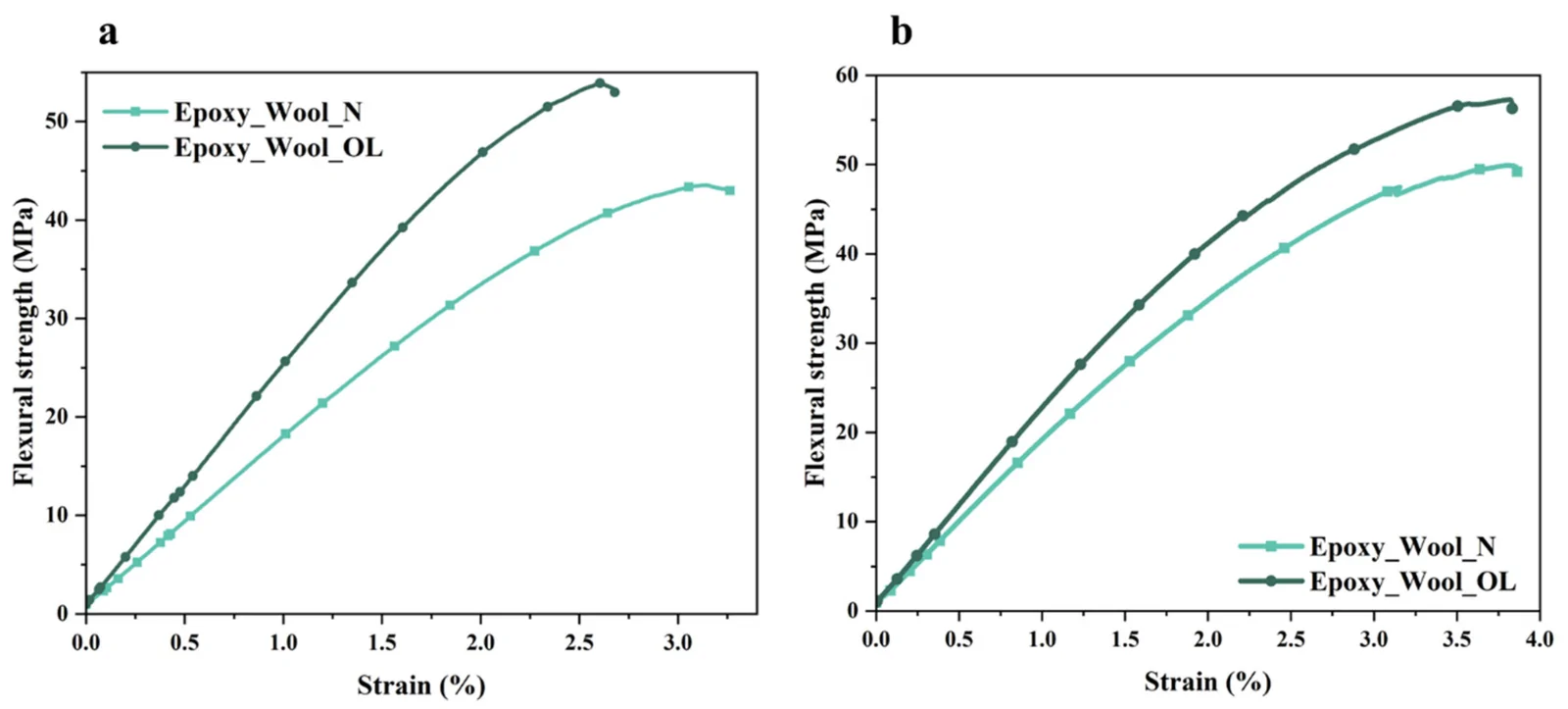
Representative flexural stress–strain curves obtained from three-point bending tests of Epoxy_Wool_N and Epoxy_Wool_OL laminates: (**a**) dry state (before water immersion) and (**b**) water immersion and re-drying.

To relate water uptake to mechanical performance, flexural behavior was then evaluated in the dry state and after the immersion–drying cycle. In the dry state (Figure 2a and Table 2), Epoxy_Wool_OL exhibits a markedly improved flexural response compared with Epoxy_Wool_N with the flexural strength increasing from 43.5 ± 0.1 MPa to 52.7 ± 1.2 MPa (+21.1%), while the flexural modulus rose from 1.6 ± 0.2 GPa to 2.1 ± 0.1 GPa (+31.3%). The strain at break decreased slightly, from 3.1 ± 0.1% to 2.7 ± 0.3%. This combination of higher stiffness and strength with a modest reduction in ductility suggests a more cohesive fiber–matrix interface, where load transfer is more efficient. This interpretation aligns with literature showing that strengthening the fiber–matrix interface enhances mechanical performance in polymer composites. For example, Conzatti et al. investigated thermoplastic systems reinforced with wool fibers and assessed strategies to enhance interfacial compatibility. In polyester/wool composites, a fiber PVA pre-treatment increased the tensile modulus from ~338 MPa (untreated) to ~437 MPa (PVA-treated) at 30 wt% fiber, with small but measurable gains in yield and ultimate tensile strengths [65]. In polypropylene/wool composites, the addition of a maleated compatibilizer (PP-g-MA) promoted adhesion and reduced pull-out, resulting in higher tensile stiffness compared with neat PP matrices. However, the tensile strength remained constrained by the short effective fiber length after melt blending (0.08–0.11 mm), which was well below the critical length (L_c_ ~ 0.72 mm) [66].

After the immersion–drying cycle (Figure 2b and Table 2) the stress–strain curves reveal that both systems, Epoxy_Wool_N and Epoxy_Wool_OL, retained their structural integrity and even exhibited an enhancement of respective flexural properties after water exposure.

Quantitatively, Epoxy_Wool_N reached a flexural strength of 49.3 ± 3.7 MPa with a flexural modulus of 1.7 ± 0.3 GPa, whereas Epoxy_Wool_OL achieved higher values of 56.4 ± 1.5 MPa and 2.4 ± 0.1 GPa, respectively. Compared with the corresponding dry composites, these values represent increases of approximately 13% for the neat wool reinforced composite and 7% for the lignin-modified fabric composite.

Changes in the mechanical properties of natural fiber reinforced composites following moisture exposure and subsequent drying or moisture cycling have also been reported in the literature. For example, Lu and van Vuure [67] observed increases in the flexural strength and modulus of flax fiber composites after repeated moisture cycling, whereas Fiore et al. [45] reported substantial recovery of flexural strength following drying after salt-fog exposure. Although these studies involve different reinforcing fibers and conditioning procedures, they demonstrate that the mechanical response of natural fiber composites following environmental exposure may not be limited to reversible changes associated with moisture uptake. In the present study, the flexural tests were performed after the specimens had been dried following water immersion. Therefore, the observed increases cannot be explained solely by fiber swelling during immersion, as subsequent drying may induce fiber shrinkage and partially reverse the associated dimensional changes. In particular, the increases in flexural properties cannot be directly attributed to the closure of interfacial gaps or to improved fiber/matrix adhesion. Moisture-induced structural rearrangements in wool keratin may also be considered as a possible contributor to the observed changes in flexural properties. Previous research has shown that the physical and mechanical properties of wool fibers can be affected by treatments involving water vapor and heat, with some changes associated with reorganization of the keratin structure [68]. Consequently, the observed improvements cannot be conclusively attributed to the closure of interfacial gaps, enhanced fiber/matrix adhesion, or irreversible structural changes in the wool fibers. Dedicated investigations, including microstructural characterization of the fiber/matrix interface and structural and thermal analyses of the wool fibers before and after immersion–drying cycles, are needed to clarify the underlying mechanisms and establish their contribution to the observed changes in flexural properties.

It is worth noting that the magnitude of the observed increase in flexural properties differs depending on the reinforcement type. The composite reinforced with neat wool fibers exhibited a larger relative increase than that containing the lignin-modified fabric. This difference may be partly related to the higher water uptake observed for the untreated wool fibers, which may result in a different response to moisture exposure and subsequent drying.

### 3.3. Fracture-Surface Morphology (SEM) After Three-Point Bending

**Figure 3:**
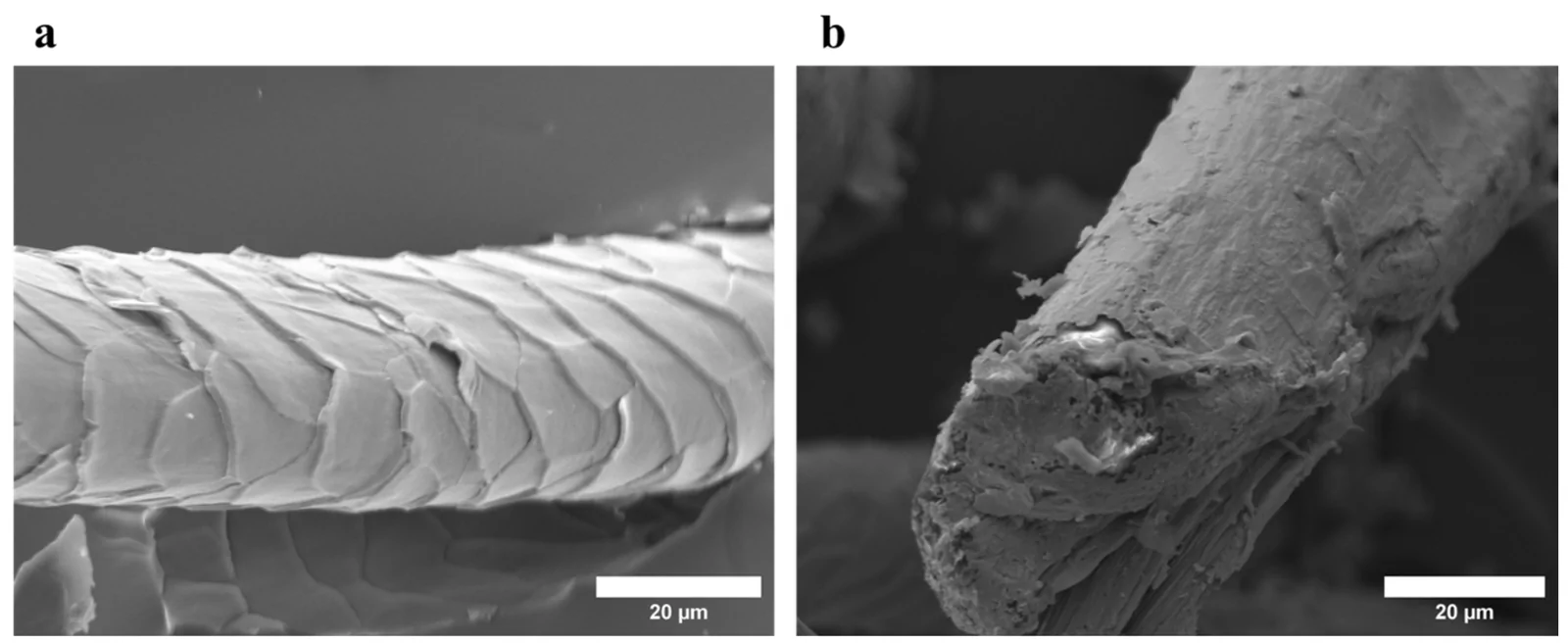
SEM fracture surfaces after three-point bending test: (**a**) Epoxy_Wool_N and (**b**) Epoxy_Wool_OL composites.

SEM analysis of the fracture surfaces after three-point bending (Figure 3) was performed to provide morphological support for the differences observed in the flexural response of the laminates. In Figure 3a Epoxy_Wool_N, the fracture surface is characterized by extensive fiber pull-out and clean, undamaged fiber cuticles indicative of interfacial debonding-dominated failure. By contrast, in Figure 3b, Epoxy_Wool_OL, fibers frequently displayed epoxy residues adhering to the cuticle, suggesting a more cohesive fiber–matrix region. These morphological features are associated with the increase in flexural modulus and strength observed for the coated laminates.

### 3.4. Short-Beam Shear (SBS) and Charpy Impact Tests

Table 3 shows that the ILSS increased from 8.6 ± 0.7 MPa for Epoxy_Wool_N to 9.3 ± 0.5 MPa for Epoxy_Wool_OL, corresponding to an ~8% gain. This modest increase suggests improved interlaminar load transfer. Similarly, lignin-coated carbon fibers in epoxy showed a ~27% increase in micromechanical IFSS, attributed to improved wetting and interactions between lignin functional groups and the epoxy network [69]. The reported increase in single-fiber adhesion is in agreement with the slight ILSS increase observed in our work at the laminate scale.

Charpy impact tests (Table 3) revealed no significant difference in impact strength between Epoxy_Wool_N and Epoxy_Wool_OL composites. Nonetheless, the higher peak force measured for Epoxy_Wool_OL points to more effective fiber–matrix load transfer provided by the lignin-based coating, in agreement with the trends observed in the quasi-static bending tests [70]. These results indicate that the effect of lignin modification is not equally pronounced across all mechanical tests. Nevertheless, when considered alongside the flexural results and the morphological evidence previously discussed, they contribute to a broader assessment of the mechanical response associated with fiber modification.

### 3.5. Low-Velocity Impact Tests and Related Damage Morphology

**Figure 4:**
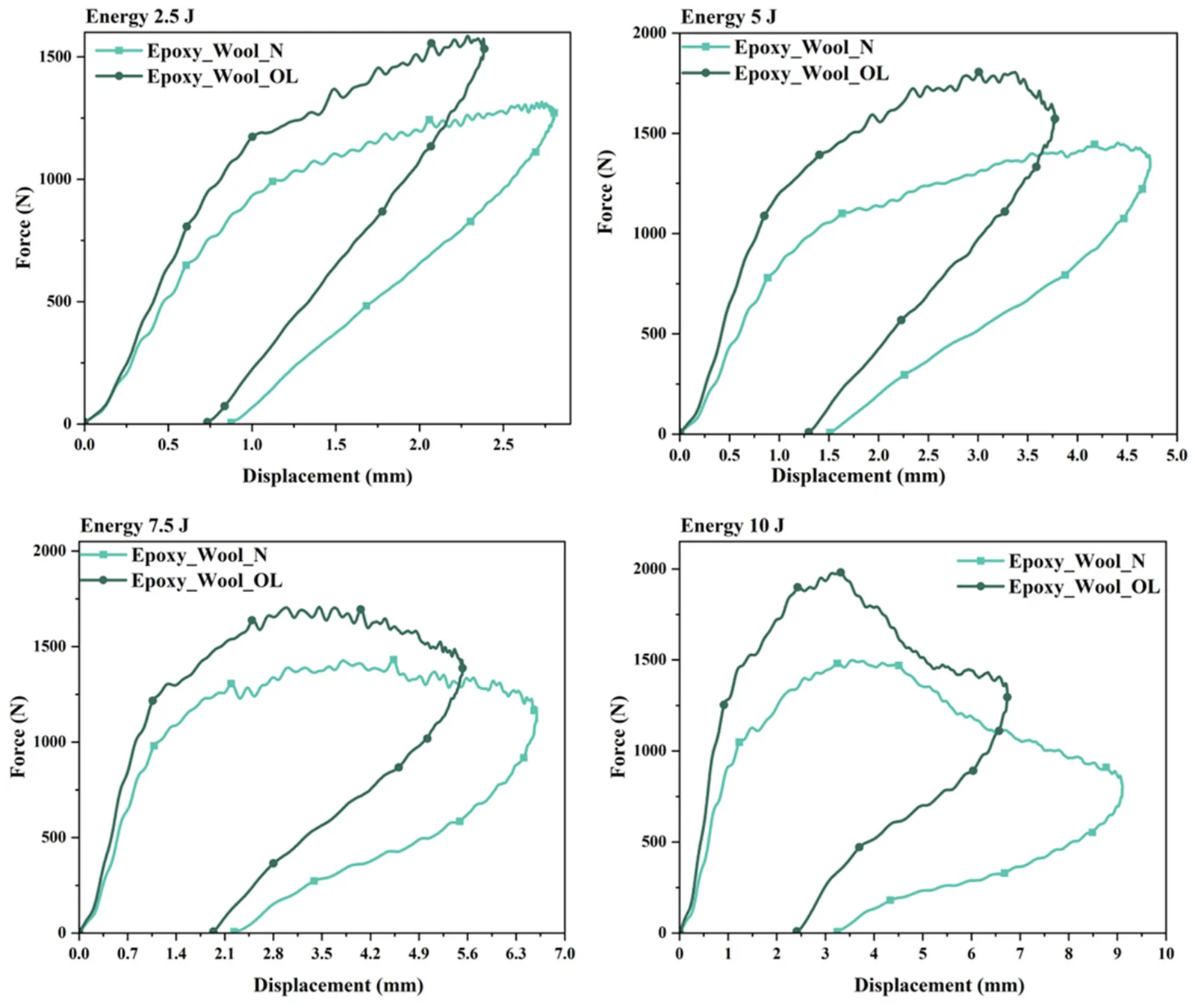
Low-velocity impact force-displacement curves for epoxy-wool laminates at nominal impact energies of 2.5, 5.0, 7.5 and 10 J.

Figure 4 shows the force–displacement curves of Epoxy_Wool_N and Epoxy_Wool_OL composites at the investigated impact energies. All curves exhibited impactor rebound without perforation. The coated laminates showed a steeper initial slope and higher peak force than the neat-wool laminates. Increasing the impact energy from 2.5 to 5 J raised the peak force by approximately 15% in both systems, while a further increase of about 20% from 5 to 10 J occurred only in the coated laminate. The increase in peak force with improved fiber/matrix interfacial effectiveness is in line with established interpretations of low-velocity impact behavior in natural fiber/epoxy laminates [71].

**Figure 5:**
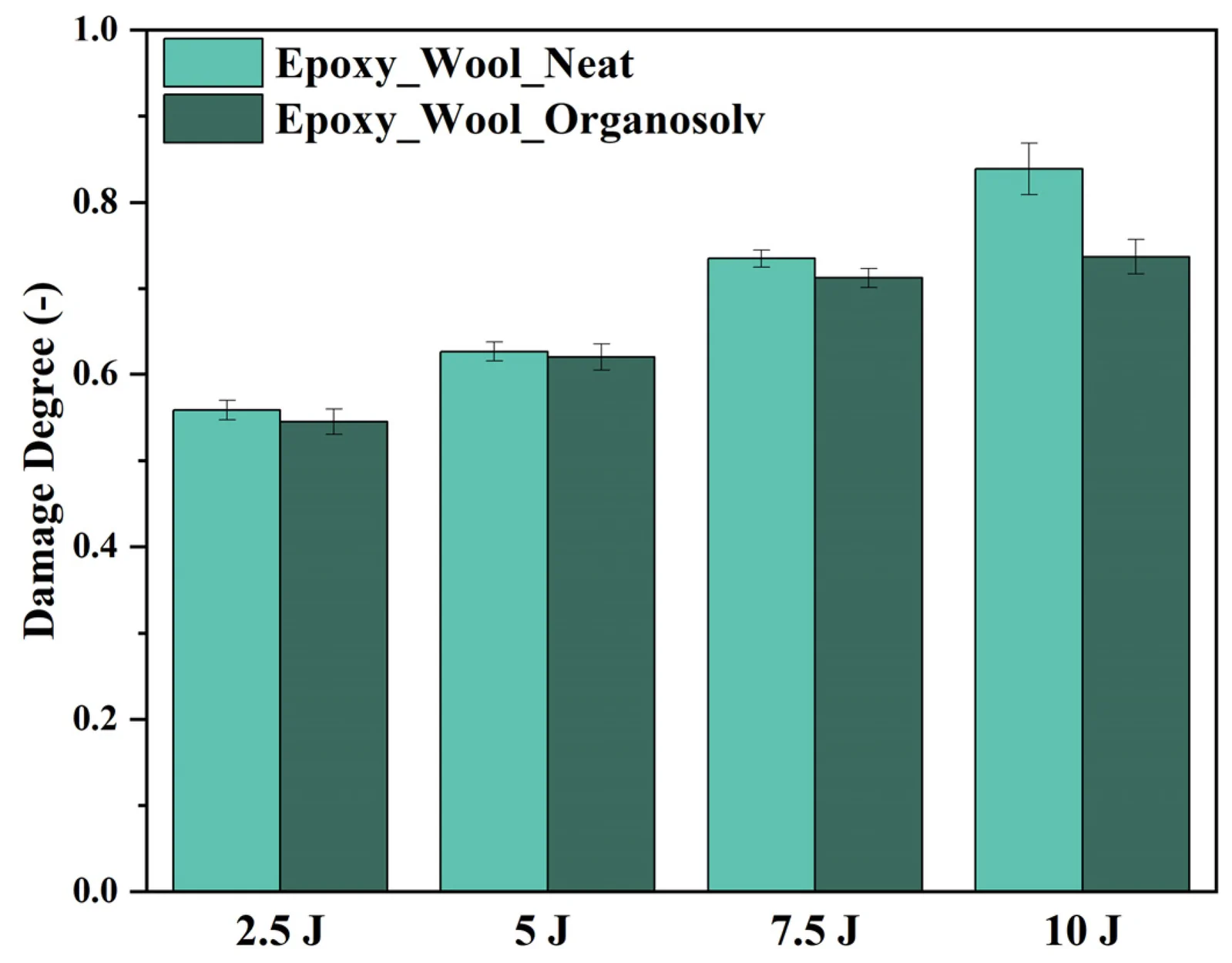
Damage degree versus incident energy for Epoxy_Wool_N and Epoxy_Wool_OL composites.

Figure 5 shows the damage degree (defined as the ratio between the absorbed energy and the impact energy) as a function of incident energy. As expected, damage increased with energy level. At a given energy, Epoxy_Wool_OL exhibited slightly lower values than Epoxy_Wool_N, with the difference most pronounced at 10 J. This trend can be attributed to the higher load-carrying capacity of the modified composites, as discussed previously.

The combination of higher peak force and lower damage degree at equal energy indicates that a smaller fraction of the input energy is dissipated through irreversible mechanisms such as matrix cracking, delamination, and fiber pull-out. This behavior may reflect a more cohesive fiber–matrix interface provided by the lignin coating. A similar phenomenon has been reported for nonwoven jute/epoxy laminates, where enhanced adhesion through chemical treatment and nanocellulose spray coating increased peak force and reduced damage at fixed energy, with concurrent improvements in compression-after-impact performance [72].

To further examine damage initiation, X-ray micro-CT was performed on specimens impacted at 2.5 J. This energy level was selected based on the force-displacement response (Figure 4) since at 2.5 J both laminates showed a comparable global damage degree and similar macroscopic appearance, whereas increasing the impact energy to 5 J led to an ~15% increase in peak force together with a more progressive plastic response. Thus, 2.5 J was considered suitable for evaluating damage initiation in the rebound-dominated regime.

**Figure 6:**
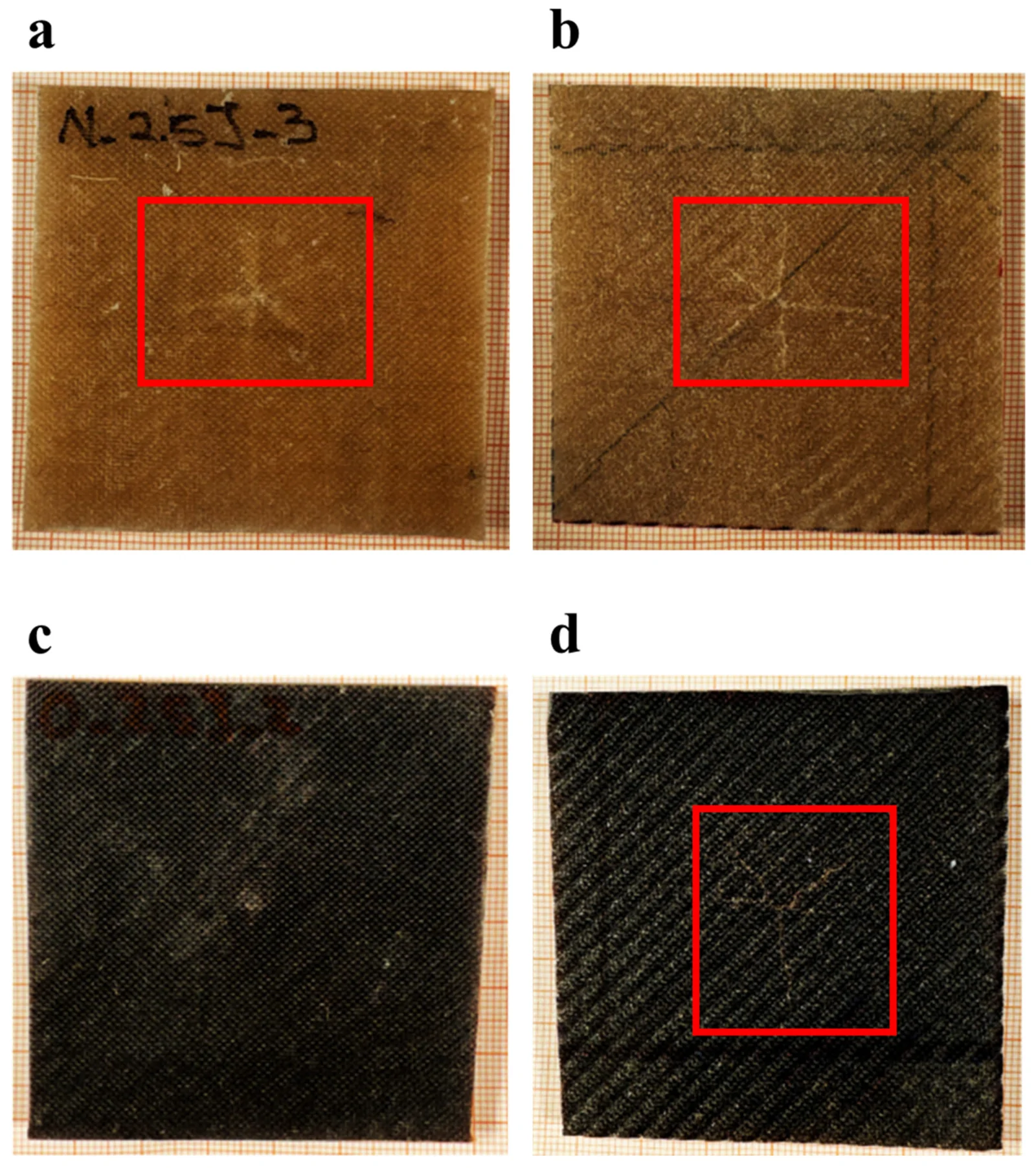
Macrophotographs of laminates impacted at 2.5 J: Epoxy_Wool_N (**a**,**b**) and Epoxy_Wool_OL (**c**,**d**). Panels (**a**,**c**) show the respective impacted faces, while panels (**b**,**d**) show the respective back faces.

**Figure 7:**
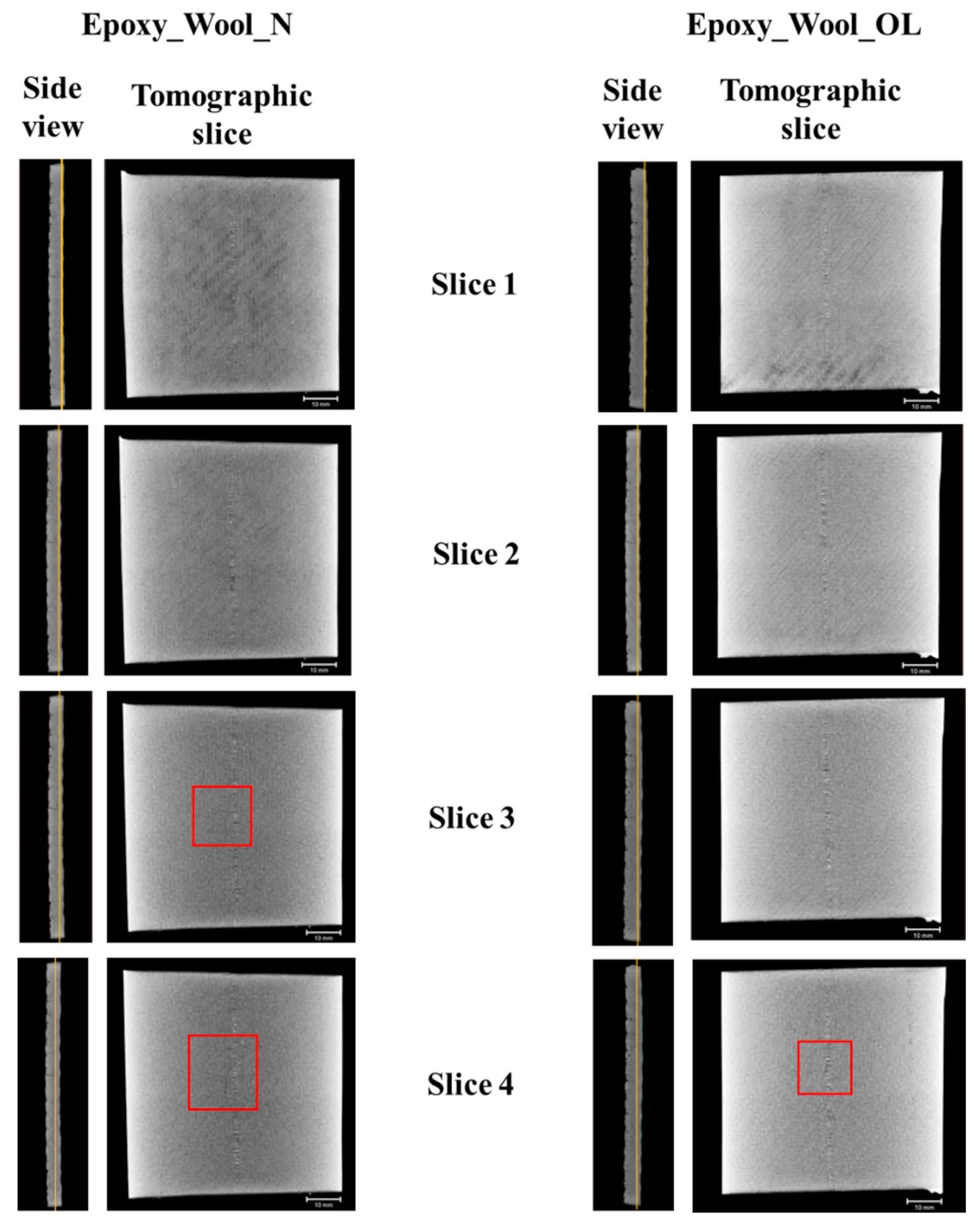
X-ray microtomography of Epoxy_Wool_N (**left**) and Epoxy_Wool_OL (**right**) laminates after low-velocity impact at 2.5 J. In each row, the narrow panel shows the side/depth view of the specimen, and the yellow line indicates the position of the corresponding tomographic slice shown in the adjacent panel. The images reveal the onset of through-thickness tensile cracking, highlighted by red boxes.

Although macroscopic inspection suggested minimal differences between the two laminates (Figure 6), the tomographic reconstructions revealed that, even at 2.5 J, the neat composite developed more pronounced through-thickness tensile cracking than Epoxy_Wool_OL (Figure 7). The first detectable damage features appeared at ~27% of the laminate thickness in Epoxy_Wool_N, compared with ~33% in Epoxy_Wool_OL, indicating a delayed/deeper onset of tensile-driven damage in the coated laminate. This trend can be explained by the bending stress state during low-velocity impact, which generates tensile stresses at the back face of the laminate. The reduced crack development in Epoxy_Wool_OL suggests that the lignin coating enhances the fiber–matrix interphase, thereby limiting early tensile crack initiation and growth.

**Figure 8:**
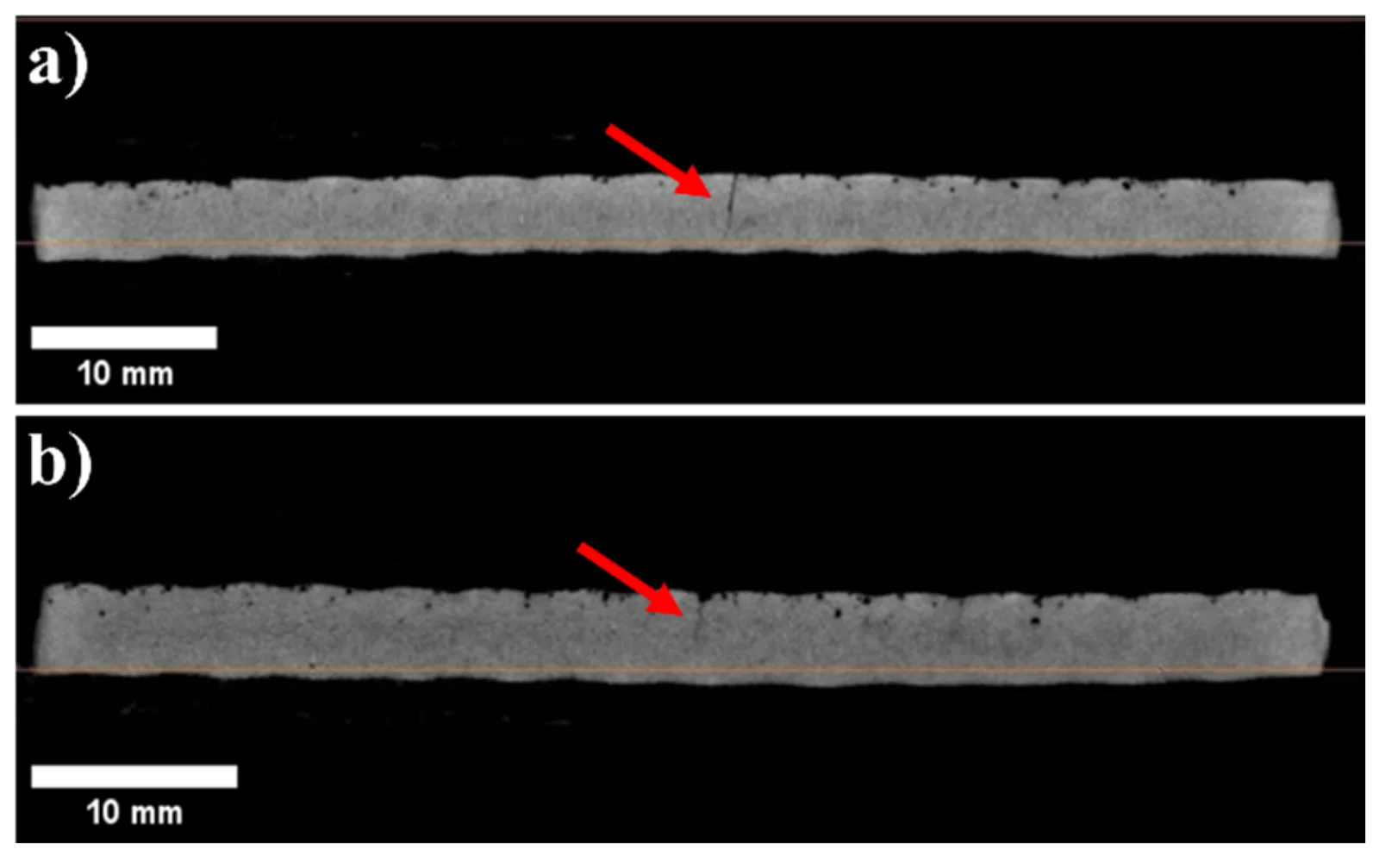
Radiographic Z-view 2D projections of laminates impacted at 2.5 J: (**a**) Epoxy_Wool_N and (**b**) Epoxy_Wool_OL. Red arrows highlight the main crack features used to measure the projected crack length.The yellow lines indicate the locations of the cross-sectional planes corresponding to the micro-CT slices shown alongside the radiographic projections.

To complement the qualitative inspection of the tomographic slices, projected crack length was measured from 2D radiographic Z-view projections (Figure 8). Although projection-based measurements integrate overlapping features through the thickness, using identical acquisition and projection settings provides a consistent basis for comparing the two laminates. Using this approach, the projected crack length was 2.73 ± 0.19 mm for Epoxy_Wool_N and 2.05 ± 0.07 mm for Epoxy_Wool_OL. The ~25% reduction in crack length indicates that the lignin-coated laminate develops more localized damage and delays through-thickness crack propagation under low-velocity impact. This behavior agrees with the tomographic observations, which show earlier tensile failure in the neat laminate, and suggests that the lignin coating improves resistance to crack growth without preventing damage entirely.

Overall, the observed improvements in interlaminar and impact-related properties, while moderate in magnitude, are consistent across quasi-static, dynamic, and impact-loading conditions, supporting the interpretation of enhanced fiber–matrix load transfer.

### 3.6. Dynamic Mechanical Analysis (DMA)

**Figure 9:**
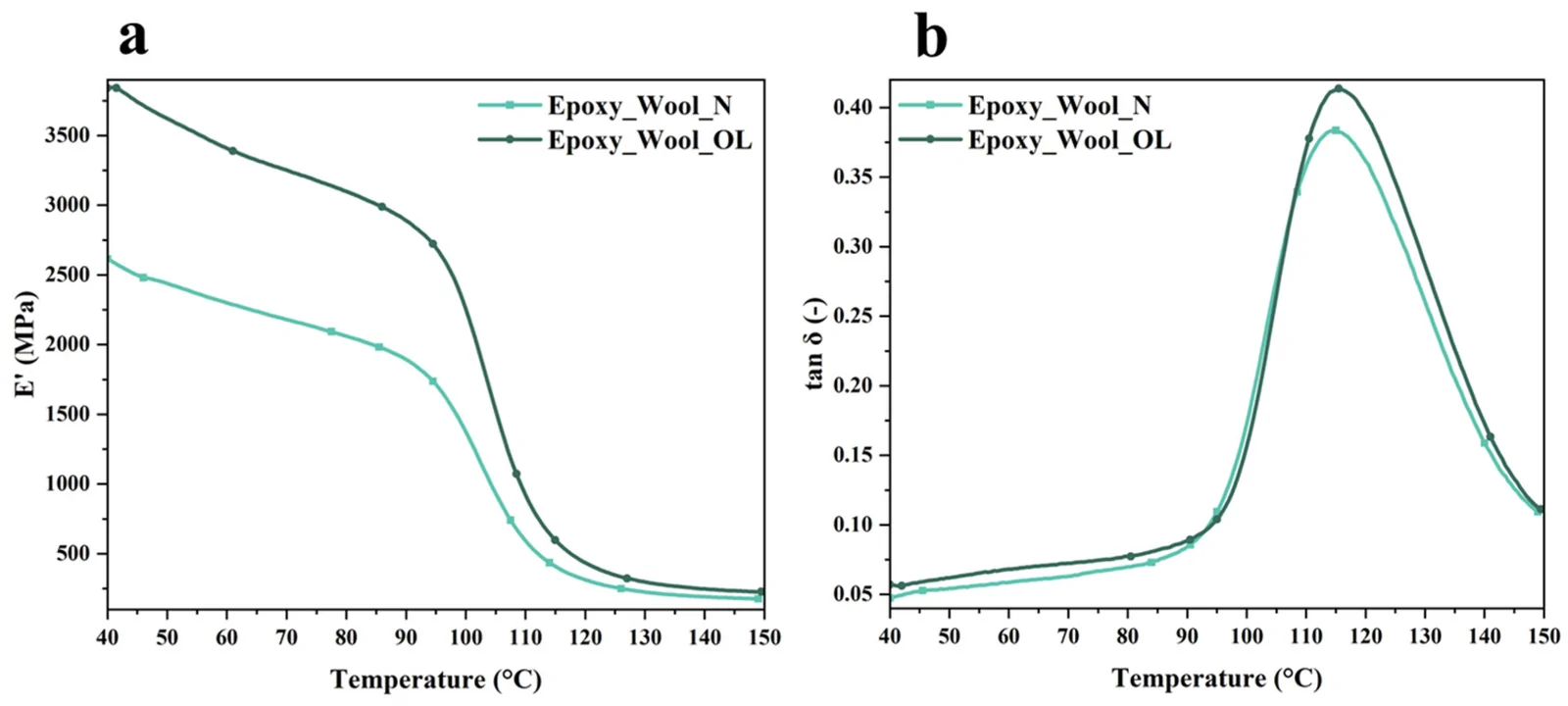
DMA results: (**a**) storage modulus (E′) and (**b**) damping factor (tanδ) as a function of temperature for Epoxy_Wool_N and Epoxy_Wool_OL composites.

Dynamic mechanical analysis (DMA) was used to assess the viscoelastic response of the epoxy-based composites. The storage modulus (E′) and the loss factor (tan δ) are reported in Figure 9. In the 40–150 °C range (Table 4), the E′ curves show a clear increase for Epoxy_Wool_OL relative to Epoxy_Wool_N. This rise in stiffness agrees with the previous results and points to stronger interfacial adhesion between the wool fibers and the epoxy matrix in the presence of the lignin-based coating [73]. The effect is evident not only in the glassy region, where the higher E′ confirms improved load transfer, but also in the transition and rubbery regions, where the coated system maintains a higher modulus. Such behavior reflects a more effective stress transfer and a constrained molecular mobility at the interphase, a trend commonly observed in natural fiber composites after surface treatments [74].

The tan δ analysis indicates that the glass-transition temperature (T_g_) remains essentially unchanged between the two composites. Nevertheless, the peak becomes slightly higher and broader in the composite reinforced with lignin-coated fibers. This change indicates a modification of the damping response around the glass transition, but cannot be directly interpreted as evidence of stronger interfacial constraint. The increase in storage modulus (E′) suggests a stiffer composite, whereas the changes in tan δ may reflect the combined influence of interfacial interactions and microstructural factors affecting energy dissipation. As reported for Sansevieria cylindrica/polyester composites [75], surface treatments can modify both stiffness and damping behavior, with the latter being influenced by factors such as interphase characteristics, fiber misalignment, broken fiber ends, matrix microcracking, and cavities associated with fiber pull-out. Accordingly, although the present results indicate changes in both the stiffness and viscoelastic response of the lignin-modified composite, further investigation would be required to determine the specific mechanisms responsible for the observed tan δ peak broadening and increase.

### 3.7. Thermal Stability (TGA)

**Figure 10:**
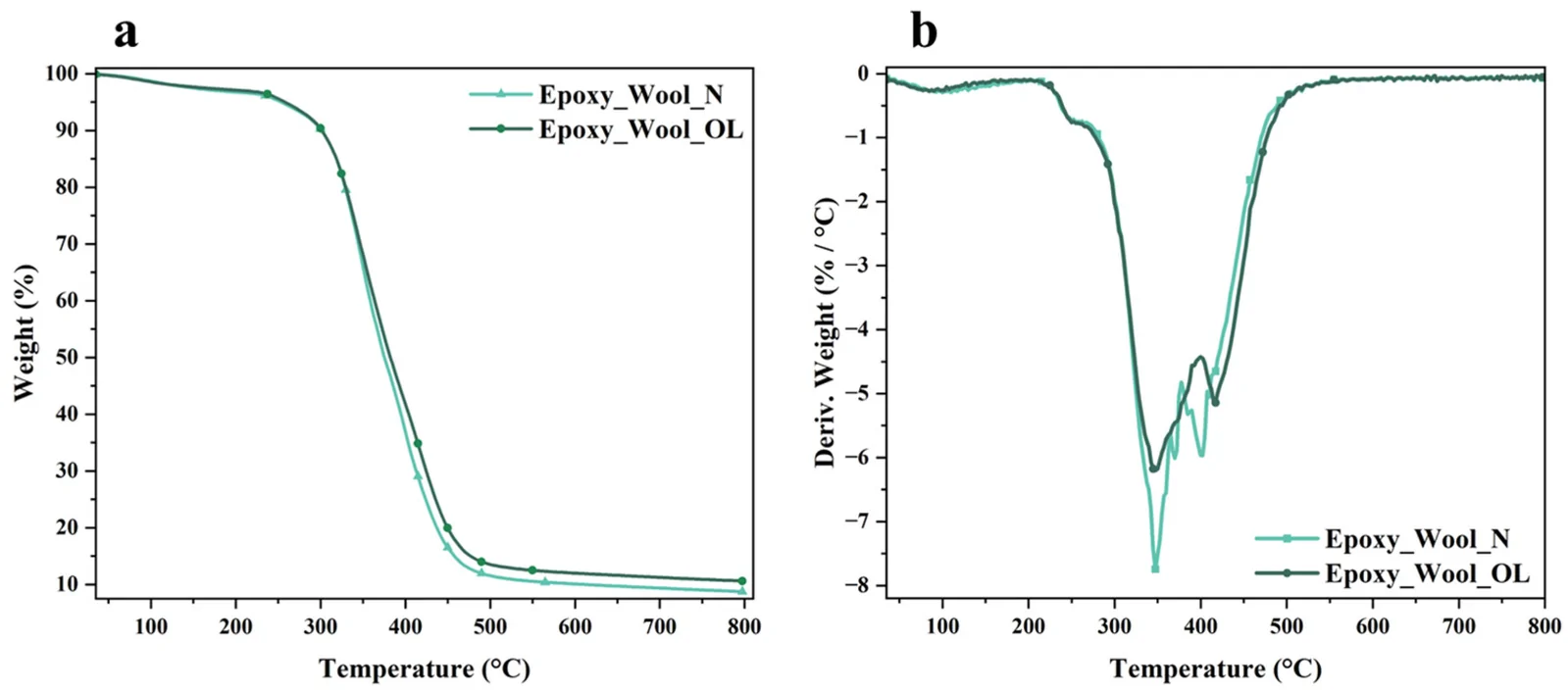
Thermogravimetric (**a**) and derivative thermogravimetric (dTG) (**b**) curves of epoxy composites reinforced with neat wool fabric (Epoxy_Wool_N) and wool fabric coated with organosolv lignin (Epoxy_Wool_OL).

Thermogravimetric analysis (TGA) was performed on epoxy composites reinforced with neat wool fabric (Epoxy_Wool_N) and with wool fabric coated with organosolv lignin (Epoxy_Wool_OL). Figure 10 reports both the weight loss and the derivative thermogravimetric (dTG) curves, allowing a direct comparison of the thermal degradation behaviors of the two composite systems. Table 5 summarizes the onset degradation temperatures at 5% (T_5_%) and 10% (T_10_%) weight losses, the temperature at the maximum decomposition rate (T_max_), and the residual mass at 800 °C. The analysis shows for both composites a multistage trend typical of epoxy resins cured with amines, characterized by an initial region of limited mass loss below 300 °C, followed by two main dTG peaks located at about 347 °C (T_max1_) and between 401 and 416 °C (T_max2_). This two-step behavior is well documented for amine-cured epoxy systems, in which the first maximum, generally between 340 and 370 °C, is attributed to the cleavage of ether linkages and aliphatic units generated during curing, whereas the second maximum, between 400 and 460 °C, corresponds to the degradation of the aromatic backbone and the formation of carbonaceous residue [76]. The shoulder visible at around 240–260 °C is consistent with the contribution of wool, whose keratin begins to degrade in this range through S–S bond and side-chain scissions, while an additional maximum at 320–340 °C is associated with the decomposition of the peptide backbone [77,78]. Consequently, the 200–400 °C interval of the composite dTG reflects the superposition of a secondary contribution from wool with the first degradation stage of the resin. The comparison between Epoxy_Wool_N and Epoxy_Wool_OL highlights two main differences: an increase in the residual mass at 800 °C (from 8.7 to 10.6 wt%) and a shift in T_max2_ from 400.7 °C to 416.2 °C (Table 5). Both effects are attributable to the presence of the lignin coating, an aromatic material known to promote char formation under inert atmosphere and to act as a condensed-phase barrier and radical trap, thereby slowing the decomposition of the network at high temperature [79]. The fact that T_5%_ and T_10%_ remain essentially unchanged (~253–258 °C; ~301 °C) indicates that the early mass losses, associated with residual moisture, weaker bonds, and the initial contribution of wool, are not significantly influenced by the lignin coating, whereas the stabilization effect becomes evident in the structural degradation stage of the resin above 400 °C. Overall, the two main peaks observed in the composites (T_max1_ ~ 347 °C; T_max2_ ~ 401–416 °C) can be attributed predominantly to the epoxy resin cured with amines, with wool contributing only a minor shoulder at lower temperature, while lignin is responsible for the increased thermal stability and higher char yield.

## 4. Conclusions

This study demonstrated that applying a lignin-based coating on wool fabrics is an effective strategy to enhance the performance of wool/epoxy composites in moist environments. Water uptake of composites decreased by nearly 30%, while the effective diffusion coefficient was reduced by about one third, confirming the hydrophobic barrier and sealing effect of the lignin coating. Thermogravimetric analysis further highlighted an increase in more than 20% in char yield and a shift in the main decomposition stage to higher temperatures, indicating enhanced thermal stability. The coated laminates exhibited marked improvements in flexural behavior, with strength and modulus increasing by more than 20% and 30%, respectively, and interlaminar shear strength rising by approximately 8%. SEM analyses confirmed reduced fiber pull-out and stronger fiber–matrix bonding. Charpy and low-velocity impact tests revealed higher peak forces (up to +20%) and lower damage degree, indicating improved load-bearing capacity and damage tolerance. Dynamic mechanical analysis showed higher storage modulus values across the 40–150 °C temperature range, reflecting more effective stress transfer within the fiber–resin matrix without altering the glass transition.

Overall, the results suggest that the lignin coating contributed to improved interfacial compatibility while providing a protective layer for the fibers. By improving fiber–matrix cohesion and limiting water accessibility at the interphase, lignin stabilizes the mechanical response of the composite under both dry and water-aged and subsequently dried states, while the improvements in thermal stability and char yield represent secondary, yet beneficial, effects. The present work should therefore be regarded as a proof-of-concept study of lignin-based surface engineering of wool fabrics for epoxy composites. Future work will address the systematic optimization of lignosulfonate dosage/add-on level and its relationship with composite performance. By simultaneously valorizing two underutilized by-products, coarse wool and lignin, this work demonstrates a viable pathway toward high-performance, durable, and circular epoxy biocomposites based on surface-engineered natural reinforcements.

## Figures and Tables

**Table 1:** Fitting parameters of the power-law and Fickian models for water absorption in Epoxy_Wool_N and Epoxy_Wool_OL laminates.

Sample	M∞ [%]	n	k [s^−n^]	D [m^2^/s]	R^2^ Power Law	R^2^ Fick
**Epoxy_Wool_N**	8.84	0.32	0.00589	5.38 × 10^−13^	0.948	0.991
**Epoxy_Wool_OL**	6.45	0.347	0.00399	3.74 × 10^−13^	0.960	0.992

Note: M∞ denotes the equilibrium moisture content, n and k are the power-law parameters (Equation (4)), D is the diffusion coefficient estimated from the short-time Fickian approximation, and R^2^ represents the coefficient of determination of the respective models.

**Table 2:** Flexural properties of wool/epoxy laminates before and after water immersion and re-drying.

	Before Water Immersion		After Water Immersion and Re-Drying	
	Flexural Strength (MPa)	Flexural Modulus (GPa)	Flexural Strength (MPa)	Flexural Modulus (GPa)
**Epoxy_Wool_N**	43.5 ± 0.1	1.6 ± 0.2	49.3 ± 3.7	1.7 ± 0.3
**Epoxy_Wool_OL**	52.7 ± 1.2	2.1 ± 0.1	56.4 ± 1.5	2.4 ± 0.1

**Table 3:** Interlaminar shear strength (short-beam shear, SBS) and Charpy impact results for Epoxy_Wool_N and Epoxy_Wool_OL laminates.

	Interlaminar Shear Strength (MPa)	Impact Strength (kJ/m^2^)	Peak Force (N)
**Epoxy_Wool_N**	8.6 ± 0.7	5.2 ± 0.4	306.4 ± 44.8
**Epoxy_Wool_OL**	9.3 ± 0.5	5.7 ± 1.4	452.5 ± 71.8

**Table 4:** Storage modulus (E′) values at different temperatures of Epoxy_Wool_N and Epoxy_Wool_OL composites.

	E′ (MPa)				T_g_ (°C)
	at 40 °C	at 75 °C	at 110 °C	at 150 °C	
**Epoxy_Wool_N**	2669.3 ± 78.4	2177.7 ± 57.7	590.7 ± 3.1	176.0 ± 19.8	115.0 ± 0.4
**Epoxy_Wool_OL**	4058.0 ± 308.3	3371.0 ± 274.4	917.0 ± 8.5	224.0 ± 2.8	115.9 ± 0.5

**Table 5:** Thermal parameters of epoxy-wool composites: onset degradation temperatures at 5% (T_5%_) and 10% (T_10%_) weight loss, maximum decomposition temperatures (T_max1_ and T_max2_), and residual mass at 800 °C.

	Residue	T_max 1_	T_max 2_	T_5%_	T_10%_
	(wt%)	(°C)	(°C)	(°C)	(°C)
**Epoxy_Wool_N**	8.7	347.1	400.7	253.3	300.9
**Epoxy_Wool_OL**	10.6	346.9	416.2	258.0	301.5

## Data Availability

The raw data supporting the conclusions of this article will be made available by the authors on request.

## Supplementary Materials

The following supporting information can be downloaded at https://www.mdpi.com/article/10.3390/polym18212756/s1, Figure S1: FT-IR spectra of Wool_N and Wool_OL; Figure S2: SEM micrographs of (a) untreated wool fabric (Wool_Neat) and (b) organosolv lignin-treated wool fabric (Wool_OL).
