# Supplementary material for: Interphase Engineering of Epoxy/Wool Biocomposites via Lignin Coating: Effects on Mechanical and Hygrothermal Performance
Source: Polymers (Basel). 2026 Nov 5;18(21):2756. doi: 10.3390/polym18212756 (PMC13765767; doi:10.3390/polym18212756)
Supplement: Supplementary file 1 [file polymers-18-02756-s001.zip › polymers-4610907-supplementary.pdf]

Interphase Engineering of Epoxy/Wool Biocomposites via Lignin Coating: Effects on Mechanical and Hygrothermal Performance

Alessia Pantaleoni[1,2*], Baljinder K. Kandola[2], A. Richard Horrocks[2], Irene Bavasso[1], Maria Paola Bracciale[1], Erika Palazzi[1], C.A. Fuentes[3,5], Khouloud Tilouche-Guerdelli[3], Jacopo Tirillò[1], Assunta Marrocchi[4], and Fabrizio Sarasini[1]

1 Department of Chemical Engineering Materials Environment, Sapienza University of Rome, 00184 Rome, Italy; irene.bavasso@uniroma1.it (I.B.); mariapaola.bracciale@uniroma1.it (M.P.B.); erika.palazzi@uniroma1.it (E.P.); jacopo.tirillo@uniroma1.it (J.T.); fabrizio.sarasini@uniroma1.it (F.S.)
2 Institute for Materials Research and Innovation, University of Greater Manchester, Bolton, BL3 5AB, UK; B.Kandola@greatermanchester.ac.uk (B.K.K.); A.R.Horrocks@greatermanchester.ac.uk (A.R.H.)
3 Luxembourg Institute of Science and Technology (LIST), L-4362 Esch-sur-Alzette, Luxembourg; carlos.fuentes@list.lu (C.A.F.); khouloud.tilouche-guerdelli@list.lu (K.T.-G.)
4 Department of Chemistry, Biology and Biotechnology, University of Perugia, 06123 Perugia, Italy; assunta.marrocchi@unipg.it (A.M.)
5 Department of Materials Engineering, University of Leuven (KUL), Leuven, Belgium
* Correspondence: alessia.pantaleoni@uniroma1.it

Supporting Information

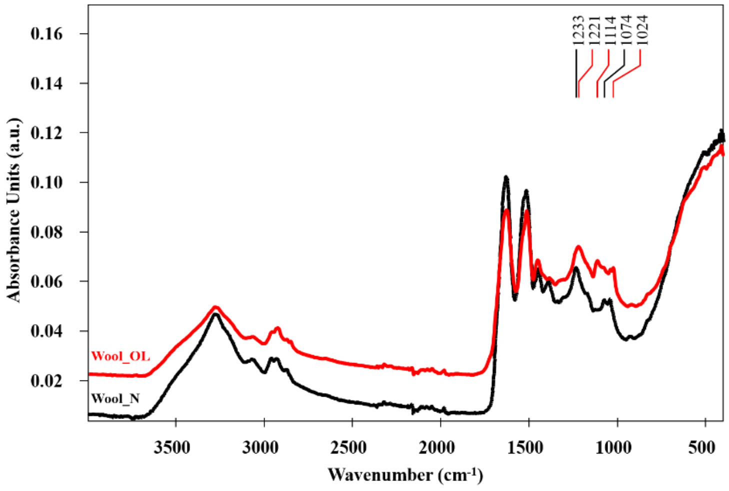

Figure S1. FT-IR spectra of Wool_N and Wool_OL.

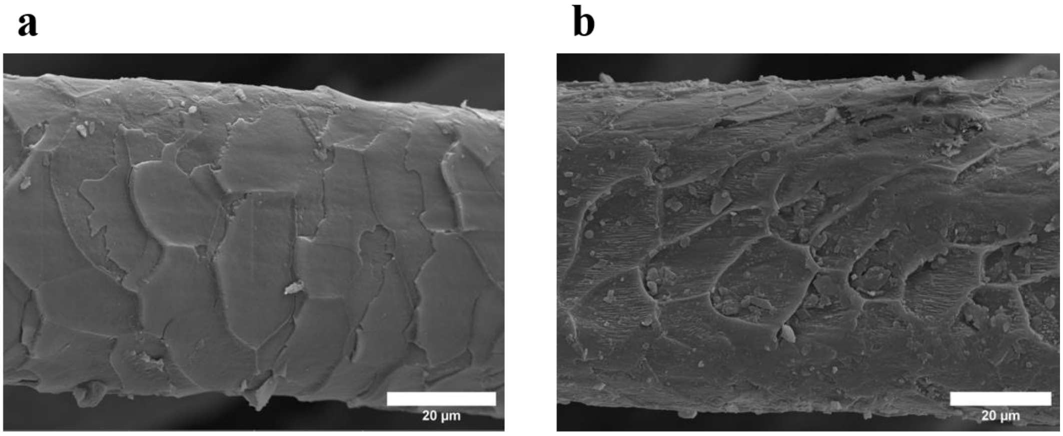

Figure S2. SEM micrographs of (a) untreated wool fabric (Wool_Neat) and (b) organosolv lignin-treated wool fabric (Wool_OL)
